# Socioeconomic Status and Associations with Nutrition in Icelandic Community-Dwelling Older Adults: Results from the AGES-Reykjavik Study

**DOI:** 10.3390/nu17203231

**Published:** 2025-10-15

**Authors:** Kristín Elísabet Halldórsdóttir, Ólöf Guðný Geirsdóttir, Ólafur Ögmundsson, Pálmi V. Jónsson, Vilmundur Guðnason, Lenore J. Launer, Hrafnhildur Eymundsdóttir

**Affiliations:** 1 Faculty of Food Science and Nutrition, University of Iceland, 102 Reykjavik, Iceland; keh13@hi.is (K.E.H.); ogg@hi.is (Ó.G.G.); olafuro@hi.is (Ó.Ö.); 2 Faculty of Medicine, University of Iceland, 102 Reykjavik, Iceland; 3 Icelandic Heart Association, 201 Kopavogur, Iceland; 4 Laboratory of Epidemiology and Population Sciences, National Institute on Aging, National Institutes of Health, Bethesda, MD 20892, USA; 5 Department of Geriatrics, National University Hospital of Iceland, 101 Reykjavik, Iceland

**Keywords:** social inequality, healthy aging, nutrition, health promotion, United Nations Sustainable Development Goals

## Abstract

**Objectives:** The growing proportion of older adults underscores the importance of healthy aging. Maintaining good nutrition and physical activity are crucial for sustaining health. However, research on aging inequalities suggests that individuals with lower socioeconomic status (SES) may be at higher risk for inadequate nutrition. The study examined associations between SES and nutrition in older community-dwelling adults in Iceland and whether the Sustainable Development Goals (SDGs) are being met in Iceland. **Methods**: Data from the AGES-Reykjavik study were used, and SES was grouped into four categories (low, medium-low, medium-high, and high), derived from education and occupation. Descriptive statistics were used to examine differences between groups, and logistic regression was used to explore associations with food and drink consumption. **Results:** Older adults with low SES were less likely to frequently consume vegetables, fruit, cod or saithe liver oil/pills, oatmeal or muesli, and cultured milk products, and they were less likely to meet nutritional guidelines. Higher SES was associated with greater alcohol intake. **Conclusions:** Older adults with low socioeconomic status are less likely to consume healthy food products than those with higher SES. As these individuals live independently, the findings highlight the need for targeted nutritional prevention and support to reduce health disparities, including potential risks such as insufficient vitamin D intake. Furthermore, the results suggest that the SDGs related to nutrition are not being met in Iceland, warranting monitoring and policy action.

## 1. Introduction

The proportion of older adults is increasing worldwide. In Iceland, older adults aged 65 and above make up approximately 15% of the total population, and according to predictions, this proportion will keep increasing [1,2].

This increase in older adults poses a challenge for health-care systems to meet the needs of this growing older population, increasing the importance of healthy aging. According to the World Health Organization (WHO), healthy aging is “a process of maintaining functional ability to enable well-being in older age” [3]. According to a WHO report on aging and health, two key behaviors influencing healthy aging are engaging in physical activity and maintaining adequate nutrition [4]. According to previous studies, poor nutritional status can predict lower quality of life among older adults [5]. However, nutritional status is affected by age-related physiological changes (e.g., poor oral health and appetite and limited mobility). It may further be related to social gradient, limiting adequate nutrition in lower socioeconomic groups [6].

However, studies concerning socioeconomic status (SES) and nutrition have been controversial. For example, a study by Hoogendijk et al. showed unadjusted baseline associations between low SES and the likelihood of malnutrition in older Spanish adults; however, after statistical correction for confounding variables (age and sex), the associations were no longer significant [7].

A Norwegian study evaluated differences in adherence to diet recommendations and found positive associations between fiber, alcohol intake, and education. It also showed a negative association between education and total carbohydrate intake [8]. In the Netherlands, individuals with low SES were found to be less likely to adhere to dietary guidelines, including fish recommendations [9]. Lack of adherence to dietary guidelines in low SES, such as due to high cost and dislike of foods, was explained in a study describing the nutritional barriers perceived by individuals [10].

The methodologies between the cross-sectional studies mentioned differ, making an absolute comparison difficult. Some studies only use education as a variable for SES, while others include either income or occupation. Most studies mentioned here have a large dataset but not all. The response rate was lacking in some studies, creating a risk for selection bias, and, most importantly, they did not adjust for important possible confounding factors, like cognitive status, social support, and multiple confounding factors predictive of health status. The association between SES groups and nutrition in older adults has not been studied in Iceland. Iceland has a low poverty rate compared to many other countries, and it is commonly thought that inequality is lower than in the rest of Europe. The Gini coefficient, which shows the income distribution within an economy, was one of the lowest in Europe for Iceland in 2022 [11].

Aging is a multidimensional process accompanied by physiological, physical, and social changes, increasing the risk for poor nutritional status and malnutrition. Since poor nutrition in older age can lead to a deterioration in various health-related conditions (i.e., sarcopenia, osteoporosis, and cachexia), understanding the underlying mechanism affecting nutritional intake is extremely important.

The United Nations’ Decade of Healthy Ageing (2021–2030) sets out to evoke changes to achieve the Sustainable Development Goals (SDGs) and healthy aging goals [12]. Goals have been set to reduce inequalities, end poverty and hunger, achieve food security, improve nutrition, and ensure quality education for all (SDGs 10, 1, 2, and 4). It is essential to understand inequality as it relates to behavior in older adults and, consequently, identify areas that can be addressed by changes in policy, recommendations, or interventions to reach the SDGs.

Iceland provides a particularly compelling setting for this investigation, since the country has one of the lowest Gini coefficients in Europe, a strong social welfare system, and a long-standing cultural tradition of regular fish consumption. These features might be expected to buffer against nutritional inequality; however, whether socioeconomic disparities in dietary habits and related nutritional status persist in such a context remains unknown.

Therefore, this study aims to examine the association between SES and nutrition among Icelandic community-dwelling older adults using data from the AGES-Reykjavik study, which has a large dataset (n = 5764) and offers the possibility of adjusting for many possible confounding variables. More specifically, the study aims to research general adherence to guidelines, isolate the effect of SES, and explore whether the SDGs are being met in Iceland.

## 2. Materials and Methods

### 2.1. Study Design and Participants

Data from the AGES-Reykjavik study examined the association between socioeconomic status and food consumption in Icelandic older adults [13]. The AGES-Reykjavik study examined participants (n = 5764) of the Reykjavik study, a prospective cardiovascular study. The study initially included men and women living in Reykjavik in December 1966, who were born between 1907 and 1937. The AGES-Reykjavik study evaluated risk factors for disability and disease in aging by examining environmental and genetic factors and their interactions. Data collection was carried out during three visits from 2002 to 2006. In this study, data from the baseline assessment are used. Data used in this study include results from a retrospective questionnaire concerning lifestyle factors, social support, and a baseline assessment of cognitive function, as well as blood samples for evaluating serum concentration of 25-hydroxy-vitamin D (25(OH)D).

### 2.2. Food Consumption Assessment

Food consumption was assessed with a validated self-report retrospective food-frequency questionnaire [14]. Participants were asked how often they consumed particular food items (vegetable salad or other kinds of raw vegetables, boiled or fried vegetables, fresh fruit, cod or saithe liver oil or liver oil pills, whole-wheat bread or another kind of coarse grain, oatmeal or muesli, rye bread or flatbread made from rye, cultured milk products, skyr (an Icelandic milk-curd product) or milk gruel or porridge, milk (whole milk, low-fat milk, skim milk, and 1% low-fat milk), corned meat sausage or another kind of salted/smoked meat, and blood sausage or liver sausage) or meals (hot meal as a main meal, meat or a ground-meat dish for a main meal, and fish or a fish dish for a main meal), with possible answers being (1) never, (2) less than once a week, (3) 1–2 times a week, (4) 3–4 times a week, (5) 5–6 times a week, (6) daily, and (7) more than once a day. Participants were further asked about their coffee consumption, including the following answers: (1) never, (2) less than one cup a day, (3) 1–2 cups a day, (4) 3–4 cups a day, (5) 5–6 cups a day, and (6) 7 or more cups a day. Answers for foods/meals were categorized into (1) 2 times a week or less, (2) 3–4 times a week, or (3) daily or more than once a day. Answers for coffee were categorized into (1) 0–2 cups a day, (2) 3–4 cups a day, or (3) 5 or more cups daily. Finally, alcohol consumption was assessed by self-report with the following questions: “do you drink alcoholic beverages now” (yes/no) (used in binary logistic regression); “how often do you drink alcoholic beverages now”, (1) daily, (2) 2–3 times a week, (3) 1 time a week, (4) 2–3 times a month, (5) 1 time a month, or (6) less than 1 time a month; and “on occasions when you drink, how much do you drink”, (1) 1 drink, (2) 2 drinks, (3) 3 drinks, or (4) 4 drinks or more. Answers were recoded to g/week (for use in descriptive statistics).

### 2.3. Physical Activity Assessment

A self-assessment questionnaire was used to assess physical activity. Participants were asked how often they had engaged in moderate-to-vigorous and, separately, light physical activity in the past 12 months. Possible answers included (1) never, (2) rarely, (3) weekly but less than 1 h per week, (4) 1–3 h per week, (5) 4–7 h per week, and (6) more than 7 h per week. Answers were categorized into (1) less than 1.5 h/week or (2) 1.5 h/week or more.

### 2.4. Socioeconomic Status

Socioeconomic status (SES) was based on questions regarding the highest level of education and occupational scale. Four escalating categories of SES were constructed, as follows: low SES, middle-low SES, middle-high SES, and high SES. This categorization follows research on class awareness in Icelanders [15].

The education levels used when constructing the socioeconomic status variable were (1) primary, (2) secondary, (3) college, and (4) university.

**Figure 1:**
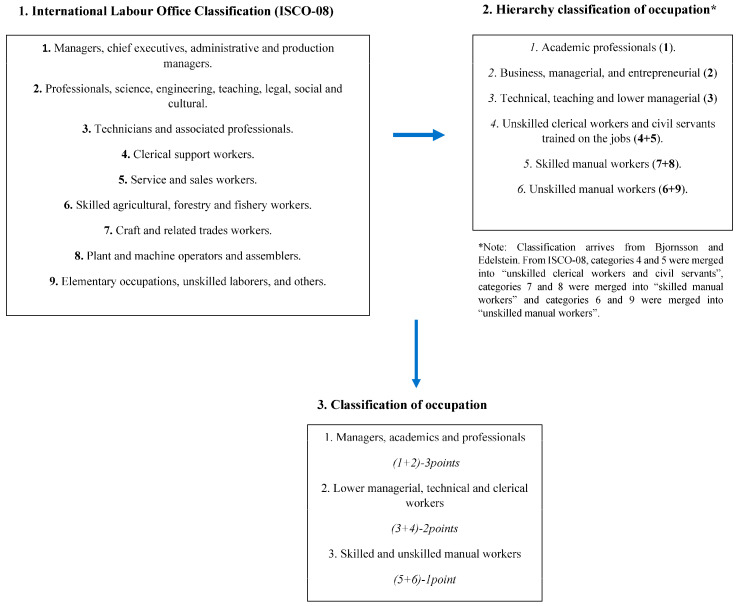
Scaling of occupation. International Labor Office Classification: merged based on social context of Icelandic society. Adopted from Bjornsson and Edelstein: merged into a three-level occupational scale.

The occupational scale was based on questions concerning the most long-held occupation, which was categorized into nine groups according to the International Labor Office Classification (ISCO-08) (Figure 1) [16]. Occupational groups were simplified by merging them into a 6-level scale, based on previous studies on the occupational scale [17], and, finally, merged into a 3-level scale, as shown in Figure 1.

### 2.5. Serum 25OHD Measurements

Fasting blood samples were drawn at the Icelandic Heart Association Laboratory. The samples were stored at −70 °C, and 25OHD measurements were performed in batch using the Liaison chemiluminescence immunoassay (DiaSorin Inc., Stillwater, MN, USA). Measured serum 25(OH)D levels were standardized in accordance with the international Vitamin D Standardization Program (VDSP).

### 2.6. Covariates

The following covariates were used in the models: age, sex, light physical activity, moderate-to-vigorous physical activity, smoking status (never, previous, or current), alcohol consumption (g/week), number of medications (evaluated by a self-report questionnaire), BMI (kg/m^2^), living alone, and cognitive status. The selection of covariates was based on previous studies [18,19].

BMI (kg/m^2^) was calculated, where weight and height were measured at baseline.

Living alone was determined by marital status answers. Participants who answered married/cohabiting were considered living with others, while those who answered widowed, divorced, or single as living alone.

### 2.7. Cognitive Function

Mild cognitive impairment (MCI) was defined as deficits in memory or one other domain of cognitive function or deficits in at least two cognitive domains without affecting activities of daily living or crossing the threshold for dementia. Determination of dementia was carried out in a three-step assessment, which has been previously described in more detail in other studies [20].

### 2.8. Exclusion Due to Missing Values

**Figure 2:**
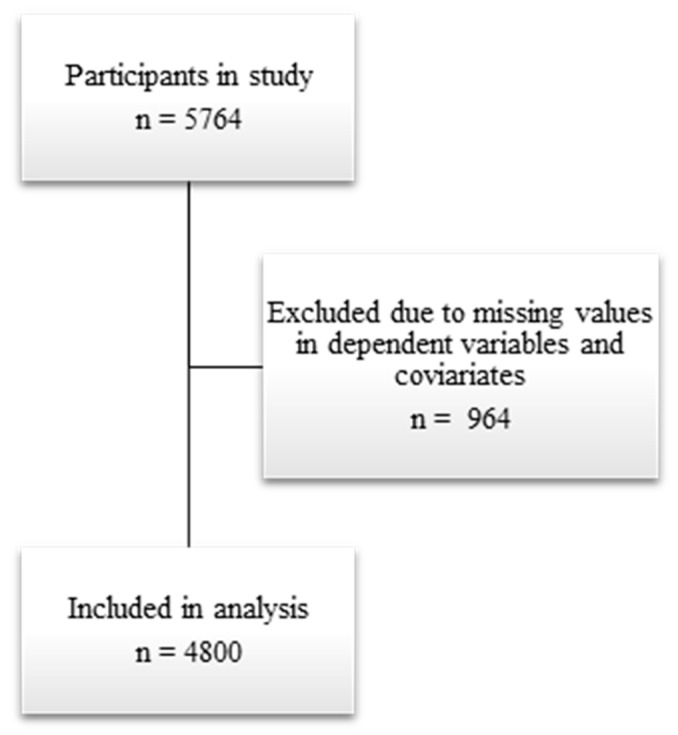
Exclusion of participants in the analysis due to missing values.

Participants with missing values in dependent variables and covariates were filtered out, as shown in Figure 2.

### 2.9. Statistical Analysis

Statistical analysis was performed with IBM SPSS Statistics 29.00.

Demographics, lifestyle, BMI (kg/m^2^), 25OHD (nmol/L), cognitive status, and number of medications were used to describe the characteristics of the participants. Distribution was described with the mean ± standard deviation (SD) for normally distributed data, for non-normal with median and percentiles (10 and 90), and percentages for categorical variables. Statistical differences between groups were tested with the Pearson chi-square (categorical variables), one-way ANOVA (normal and continuous variables), and Kruskal–Wallis tests (non-normal and continuous variables).

Associations between food consumption and socioeconomic status were evaluated with binary logistic regression. Food variables were dichotomized into daily consumption or more vs. less than daily consumption. Daily consumption was chosen because it is closest to the Nordic recommendations [21]. Coffee intake was recorded in cups per day and dichotomized as ≥5 (high) vs. 4 or less (low to moderate), based on both the cohort distribution and established caffeine safety thresholds. Over half of the participants reported consuming ≥3 cups/day, and the cut-point aligns with the 400 mg/day upper limit for caffeine, approximately equivalent to 4 cups of coffee [22]. Alcohol was dichotomized into consuming alcohol currently vs. not consuming alcohol currently.

Model 1 corrected for age and sex, and model 2 corrected additionally for health status and lifestyle (BMI, smoking, light physical activity, moderate-to-vigorous physical activity, alcohol consumption (g/week), and number of medications). Model 3 also corrected for social support, which was defined as living with others or alone. Finally, in model 4, cognitive status was added. The results are presented as the odds ratio (OR) with the 95% confidence interval, and statistical significance was assumed at a *p*-value < 0.05.

## 3. Results

Table 1 displays characteristics of the analyzed sample included in the study. Significant differences were observed in most characteristics between SES groups. The mean age of the sample was 76.5 ± 5.5 years, and most participants were female. Most participants had a low SES, or 44.2%, 25.6% had a medium-low SES, 13.7% had a medium-high SES, and 16.5% had a high SES. Participants in the lower-SES groups demonstrated a less-favorable lifestyle profile at baseline, including lower physical activity and lower serum 25-hydroxyvitamin D concentrations. Adjustment for cod-liver oil intake in the supplementary analyses did not meaningfully alter the observed differences in vitamin D levels across SES groups.

Table 2 shows the frequency of food and drink consumption by SES. Significant differences were observed in the frequency of consumption of a hot meal, meat main meal, raw vegetables, boiled or fried vegetables, fresh fruit, cod or saithe liver oil or pills, whole-wheat bread, oatmeal or muesli, cultured milk products, coffee, and alcohol between SES groups. Coffee consumption differed significantly by SES (*p* < 0.001). High intake (≥5 cups/day) was reported by 16.4% of participants overall, most frequently among those in the lowest SES group (18.9%) and least among the highest (13.7%), showing a clear inverse gradient.

Table 3 and Table 4 display the results from the binary logistic regression models examining the association between SES and food consumption. Individuals with lower socioeconomic status were significantly less likely to report daily consumption of raw vegetables, fried or boiled vegetables, fruit, cod or saithe liver oil or pills, oatmeal or muesli, and cultured milk products compared to those with high socioeconomic status. Individuals of low SES were, however, 44% more likely to drink five or more cups of coffee per day (OR: 1.44; 95% CI: 1.13–1.85, *p* = 0.004) and 57% less likely to currently consume alcohol (OR: 0.43; 95% CI: 0.35–0.52, *p* < 0.001) compared to high SES. Socioeconomic status did not predict the likelihood of consuming a hot main meal daily, meat as a main meal, or daily consumption of whole-wheat bread, fish, or coarse grain. Further analysis of specific processed meat items indicated that individuals with low SES were more likely to consume corned or smoked meat at least twice per week (adjusted OR: 2.32; 95% CI: 1.91–2.82, *p* < 0.001) and liver or blood sausage three or more times per week (adjusted OR: 2.21; 95% CI: 1.23–3.98, *p* = 0.008).Overall, statistical correction for covariates did not change the associations between consumption of food or drinks, even though for some items (coffee and oatmeal or muesli), the odds ratio was attenuated by the covariates in model 2.

Stratified analyses were conducted separately by age and by sex. Participants in the lowest SES group (compared to high SES) and ≤75 years had 75% higher odds (OR = 1.75; *p* < 0.001) of consuming >5 cups of coffee per day, while for the age group >75, it was not significant. When analyses were stratified by sex, men in the lowest SES group had approximately 60% higher odds of high coffee consumption (>5 cups per day) compared with men in the highest SES group (OR = 1.60; *p* < 0.001), whereas the association was not significant among women. The SES–diet associations for other food items remained consistent across age and sex groups.

## 4. Discussion

This study adds to the limited studies on the association between socioeconomic status and food consumption in Iceland. The results show that individuals of low socioeconomic status are less likely to consume vegetables and fruit than those of high SES. The consumption of cod or saithe liver oil or liver oil pills, which we speculate is the most abundant food source of vitamin D in Iceland for this age group, was and still is recommended for daily intake [23] and was different among the SES groups. Individuals of low SES were less likely to consume cod or saithe liver oil or liver oil pills daily. Although some previous studies did not observe significant differences in serum 25-hydroxyvitamin D [25(OH)D] concentrations across SES groups [9], our analyses are consistent with several studies indicating that older adults in lower-SES groups have lower serum 25(OH)D concentrations [24,25]. Moreover, concentrations remained significantly lower in the two lowest SES groups, even after adjusting for cod-liver oil use, suggesting that factors beyond this specific supplement contribute to the observed disparity. One potential explanation is differential sun exposure, as individuals in higher-SES groups may have greater opportunities to travel to southern latitudes, where sunlight is more abundant and cutaneous synthesis of vitamin D is facilitated. Additionally, heightened public awareness and national health initiatives promoting vitamin D intake around the time of the AGES-Reykjavík study’s initiation (2002) may have influenced supplement use behavior disproportionately across SES strata. This disproportionate response may also reflect differences in health literacy, which is more prevalent among individuals in higher-SES groups, potentially contributing to greater uptake of health recommendations [26]. Notably, our study did not identify significant SES-related differences in fish consumption, which contrasts with previous findings from a Dutch cohort [9]. This may partly reflect the historically strong cultural tradition of fish consumption in Iceland, particularly among older adults, where regular intake of various fish species has long been common regardless of individual circumstances. Fish is also a dietary source of vitamin D, especially salmon and trout, contributing to a more equal mean variance of serum 25(OH)D among participants. Other likely affordable food sources for individuals of low-socioeconomic status that include vitamin D are haddock (0.94 µg/100 g) and liver sausage (0.14 µg/100 g) [27]. However, it should be noted that our study shows information specifically on cod-liver oil intake not on total vitamin D supplementation. Thus, we can only speculate that broader supplement use and differential sun exposure may have contributed to differences in vitamin D levels among the participants. Future studies should investigate the long-term associations between SES and serum vitamin D levels.

While a higher frequency of meat consumption was observed among individuals with low SES, adjusted regression analysis did not show a significant association between SES and daily meat consumption overall. However, further examination of specific processed meat items revealed a pattern of greater intake among individuals with low SES. This may reflect differences in food cost, availability, or cultural dietary preferences, particularly regarding affordable products such as corned, smoked, or blood-based sausages.

Our study examines one underlying behavior related to health and disease in older age, and we found a significant social gradient in nutritional choices among the AGES-Reykjavík participants. Even though not confirmed in the current study, unfavorable nutritional choices in the lowest SES group are likely to negatively impact health over a more extended period of time. For instance, in our study, participants with low SES were more likely to consume coffee (over 5 cups daily) and have lower consumption of cultured milk products, presumably increasing their risk for osteoporosis [28,29]. Stratified analyses further identified a vulnerable subgroup of men under 75 years with low SES, who had higher odds of heavy coffee consumption. This may reflect behavioral or psychosocial factors, such as occupational stress or lifestyle habits more common among men in this age and socioeconomic group, which could further amplify the potential adverse effects of low dairy intake on bone health. Previous research has shown that sarcopenia and frailty are associated with poor nutritional choices and lower levels of vigorous physical activity among individuals with lower socioeconomic status [29]. Therefore, further longitudinal studies addressing the long-term consequences of poor nutrition based on socioeconomic status are needed.

Compared with other Nordic countries, the socioeconomic patterning observed in Iceland is broadly consistent with regional trends. Studies from Nordic countries, particularly Denmark and Finland, have shown that individuals with lower education or income consume fewer fruits, vegetables, and whole-grain products than those with higher socioeconomic status [30,31]. Although Iceland’s national dietary recommendations align well with the Nordic Nutrition Recommendations 2023 [32], the persistence of socioeconomic gradients in key food groups and serum 25(OH)D levels indicates that nutritional inequalities remain comparable to those in neighboring welfare states, despite Iceland’s relatively low income inequality. Notably, differences in serum 25(OH)D levels persisted even after adjustment for cod-liver oil intake, suggesting that broader lifestyle and behavioral factors, such as sun exposure, knowledge and appropriate use of supplements (nutrition literacy), and travel habits, may contribute. The absence of SES differences in fish intake, which likely reflects the cultural embeddedness of fish consumption in Iceland, underscores that social disparities in other food groups are shaped by factors beyond traditional dietary norms. Together, these findings indicate that even in highly developed welfare societies, cultural, behavioral, and cognitive factors, such as health literacy and food choice autonomy, continue to shape dietary inequalities beyond what can be explained by economic disparity alone.

Iceland offers a distinctive context for examining socioeconomic inequalities in nutrition among older adults. Despite the country’s low Gini coefficient, comprehensive welfare system, and cultural tradition of frequent fish consumption, our findings indicate that socioeconomic status continues to shape dietary patterns and nutritional status. This study challenges the common perception that Iceland, as a low-inequality society, is largely immune to social gradients in health, demonstrating that socioeconomic disparities in nutrition persist even within a highly equitable welfare context. This persistence of disparity within a low-inequality setting underscores that welfare structures alone do not eliminate social gradients in health behavior and highlights the importance of addressing underlying determinants through targeted public health initiatives.

Our study‘s results indicate, contrary to belief, that there is socioeconomic inequality in nutrition and diet in Iceland. This suggests that the SDGs of ending poverty and hunger, achieving food security, and improving nutrition, as well as good health and well-being (SDGs 1, 2, and 3) are not being met in Iceland.

From a global policy perspective, these findings can be interpreted within the framework of the SDGs. Specifically, the observed socioeconomic differences in dietary habits and vitamin D status among older adults correspond to targets under SDG 2.1.1, which calls for reducing prevalence of undernourishment in all its forms, and SDG 10.3, which seeks to ensure equal opportunities and reduce inequalities of outcome. The persistence of nutritional disparities within Iceland—despite its low national income inequality and strong welfare infrastructure—suggests that progress toward these goals remains incomplete. Continued surveillance of dietary inequality in older populations will be essential for assessing national progress toward SDG-related indicators and for designing interventions that support balanced nutrition and healthy aging.

Health literacy is now recognized as an important modifiable factor in improving health behaviors. Therefore, improving health literacy could counteract the socioeconomic inequality in nutritional choices seen in our study. Studies have found that health literacy and the high cost of healthier food options are underlying factors that may explain socioeconomic inequality in health [33,34].

To meet the SDGs, systems must be set in place to encourage individuals of lower socioeconomic status to consume more vegetables, fruit, and coarse-grain products. Lowering costs and improving health literacy are essential to promote the consumption of healthier food options and to achieve the SDGs in Iceland and, consequently, healthier aging [35].

To address these disparities, policy actions should combine structural and educational approaches. Financial incentives or subsidies for fruit, vegetables, and whole-grain products could improve affordability, while integrating nutrition and health literacy into primary care may enhance dietary guidance for older adults. Such measures could reduce socioeconomic inequalities in diet quality and support progress toward the health- and nutrition-related Sustainable Development Goals in Iceland.

Future studies should analyze the effects of poor nutrition on health and disease based on SES group.

## 5. Conclusions

This current study shows that individuals of low socioeconomic status are less likely to consume healthy food products often and are further from the recommended dietary guidelines than individuals with a high SES.

These findings are important for further research and indicate that policies are needed to achieve SDGs and healthy aging goals, including lowering the cost of more nutritious food options and finding solutions to increase health and nutrition literacy.

## 6. Strengths and Limitations

The current study is the first Icelandic study examining the relationship between socioeconomic status and nutrition, representing community-living older adults. Significantly, this study adds to the literature on social health inequality in aging in a Nordic country. The strength of the current study was a large sample size, comprising 4800 participants, and available information regarding health and lifestyle evaluations, enabling statistical adjustment for confounding factors.

Although not part of the original study plan, a post hoc analysis using MMSE scores (adjusted for age and education) showed no significant SES differences in cognitive function among participants included in the dietary analyses.Despite a higher prevalence of MCI and dementia in lower-SES groups, these findings suggest that differential recall bias is unlikely. It is an inherent limitation of this study, like all other cross-sectional studies, that one cannot disentangle cause and effect.

## Figures and Tables

**Table 1:** Characteristics of the study participants by socioeconomic status (education and occupation).

	All (n = 4800)	Low SES (n = 2120)	Medium-Low SES (n = 1227)	Medium-High SES (n = 659)	High SES (n = 794)	*p*-Value
Age (years) ^1^	76.5 (5.5)	77.2 (5.5)	75.6 (5.3)	76.2 (5.5)	76.0 (5.7)	<0.001
Sex (Women) ^2^	56.6% (2718)	52.4% (1110)	74.3% (912)	57.1% (376)	40.3% (320)	<0.001
BMI (kg/m^2^) ^1^	27.1 (4.4)	27.2 (4.4)	27.3 (4.5)	26.6 (4.4)	26.9 (4.2)	0.002
Smoking (current) ^2^	12.5% (599)	13.8% (292)	12.4% (152)	12.1% (80)	9.4% (75)	<0.001
MVPA ^2^						
1.5 h/week or more	30.9% (1482)	25.2% (535)	31.8% (390)	35.8% (236)	40.4% (321)	<0.001
LPA ^2^						
1.5 h/week or more	35.0% (1682)	30.7% (650)	35.3% (433)	38.1% (251)	43.8% (348)	<0.001
Living alone ^2^	40.4% (1939)	44.2% (938)	39.0% (479)	40.8% (269)	31.9% (253)	<0.001
Cognitive status ^2^						
Dementia MCI Normal	6.0% (286) 9.5% (456) 84.5% (4058)	9.0% (190) 15.8% (336) 75.2% (1594)	4.6% (57) 5.9% (73) 89.4% (1097)	2.6% (17) 3.2% (21) 94.2% (621)	2.8% (22) 3.3% (26) 94.0% (746)	<0.001
Medications (number/count) ^1^	4.13 (2.93)	4.29 (3.00)	4.10 (2.92)	3.93 (2.69)	3.90 (2.91)	0.003
Serum 25(OH)D (nmol/L) ^1^	57.21 (17.9)	56.1 (18.4)	56.6 (18)	59.1 (17.1)	59.5 (17.1)	<0.001
Serum 25(OH)D ^2^						
Deficiency (<30 nmol/L)	8.1% (391)	9.8% (207)	8.2% (101)	5.8% (38)	5.7% (45)	<0.001
Inadequate (30–49.9 nmol/L)	25.0% (1201)	26.5% (562)	25.6% (314)	22.0% (145)	27.7% (180)	
Adequate (>50 nmol/L)	66.8% (3208)	63.7% (1167)	66.2% (812)	72.2% (476)	71.7% (569)	

SES: socioeconomic status; MCI: mild cognitive impairment; MVPA: moderate or vigorous physical exercise; LPA: light physical activities. ^1^ Mean (standard deviation), one-way ANOVA (normal distribution). ^2^ Percentage (number of participants), Pearson chi-square.

**Table 2:** Nutritional consumption/factors of participants by socioeconomic status (derived from education and occupation).

	All (n = 4800)	Low SES (n = 2120)	Middle-Low SES (n = 1227)	Middle-High SES (n = 659)	High SES (n = 794)	*p*-Value
Hot main meal ^1^						
2 times a week or less	2.5% (120)	2.5% (53)	2.0% (24)	3.8% (25)	2.3% (18)	0.029
3–4 times a week Daily or more than once a day	18.8% (902) 78.7% (3778)	17.3% (366) 80.2% (1701)	21.2% (260) 76.9% (943)	19.4% (128) 76.8% (506)	18.6% (148) 79.1% (628)	
Meat main meal ^1^						
2 times a week or less	36.9% (1771)	37.4% (793)	39.6% (486)	36.6% (241)	31.6% (251)	0.008
3–4 times a week Daily or more than once a day	62.3% (2992) 0.8% (37)	61.6% (1306) 1.0% (21)	59.7% (732) 0.7% (9)	63.1% (416) 0.3% (2)	67.8% (538) 0.6% (5)	
Fish main meal ^1^						
2 times a week or less	30.0% (1441)	30.3% (642)	30.0% (368)	31.1% (205)	28.5% (226)	0.367
3–4 times a week Daily or more than once a day	68.9% (3305) 1.1% (54)	68.4% (1450) 1.3% (28)	68.6% (842) 1.4% (17)	68.4% (451) 0.5% (3)	70.8% (562) 0.8% (6)	
Raw vegetables ^1^						
2 times a week or less	52.4% (2513)	60.7% (1286)	48.3% (593)	47.2% (311)	40.7% (323)	<0.001
3–4 times a week Daily or more than once a day	35.5% (1705) 12.1% (582)	30.0% (635) 9.4% (199)	39.6% (486) 12.1% (148)	39.3% (259) 13.5% (89)	40.9% (325) 18.4% (146)	
Boiled or fried vegetables ^1^						
2 times a week or less	64.6% (3100)	71.8% (1522)	62.9% (772)	59.2% (390)	52.4% (416)	<0.001
3–4 times a week Daily or more than once a day	27.8% (1336)	22.1% (468)	29.7% (365)	32.0% (211)	36.8% (292)	
	7.6% (364)	6.1% (130)	7.3% (90)	8.8% (58)	10.8% (86)	
Fresh fruit ^1^						
2 times a week or less	27.5% (1320)	33.7% (715)	24.0% (295)	22.2% (146)	20.7% (164)	<0.001
3–4 times a week Daily or more than once a day	36.5 (1753)	34.5% (732)	38.5% (472)	39.3% (259)	36.5% (290)	
	36.0% (1727)	31.7% (673)	37.5% (460)	38.5% (254)	42.8% (340)	
Cod or saithe liver oil or pills ^1^						
2 times a week or less	31.7% (1520)	34.5% (732)	33.1% (406)	27.9% (198)	24.9% (198)	<0.001
3–4 times a week Daily or more than once a day	7.5% (358)	7.5% (160)	6.3% (77)	7.0% (46)	9.4% (75)	
	60.9% (2922)	57.9% (1228)	60.6% (744)	65.1% (429)	65.5% (521)	
Rye bread or rye flatbread ^1^						
2 times a week or less	45.2% (2169)	44.5% (944)	46.5% (570)	46.3% (305)	44.1% (350)	0.100
3–4 times a week Daily or more than once a day	29.3% (1406)	29.5% (625)	29.7% (365)	30.8% (203)	26.8% (213)	
	25.5% (1225)	26.0% (551)	23.8% (292)	22.9% (151)	29.1% (231)	
Whole-wheat bread or coarse grain ^1^						
2 times a week or less	11.6% (558)	12.5% (266)	9.7% (119)	11.7% (77)	12.1% (96)	0.049
3–4 times a week Daily or more than once a day	26.5% (1272)	25.6% (542)	29.1% (357)	27.8% (183)	23.9% (190)	
	61.9% (2970)	61.9% (1312)	61.2% (751)	60.5% (399)	64.0% (508)	
Oatmeal or muesli ^1^						
2 times a week or less	52.1% (2501)	52.9% (1121)	52.6% (646)	52.8% (348)	48.6% (386)	0.024
3–4 times a week Daily or more than once a day	17.8% (855)	18.3% (387)	18.5% (227)	17.6% (116)	15.7% (125)	
	30.1% (1444)	28.9% (612)	28.9% (354)	29.6% (195)	35.6% (283)	
Cultured milk products, skyr or milk gruel, or porridge ^1^						
2 times a week or less	34.3% (1645)	36.5% (774)	34.2% (420)	30.2% (199)	31.7% (252)	<0.001
3–4 times a week Daily or more than once a day	35.2% (1691)	36.0% (764)	35.9% (441)	35.4% (233)	31.9% (253)	
	30.5% (1464)	27.5% (582)	29.8% (366)	34.4% (227)	36.4% (289)	
Coffee ^1^						
0–2 cups a day	46.3% (2220)	42.3% (897)	48.0% (589)	49.8% (328)	51.1% (406)	<0.001
3–4 cups a day	37.4% (1794)	38.8% (823)	37.2% (457)	35.7% (235)	35.1% (279)	
5 or more cups a day	16.4% (786)	18.9% (400)	14.8% (181)	14.6% (96)	13.7% (109)	
Alcohol, g/week ^2^	3.2 (0.0, 33.0)	1.6 (0.0, 26.4)	3.2 (0.0, 33.0)	4.8 (0.0, 66.0)	8.0 (0.0, 66.0)	<0.001

SES: socioeconomic status. ^1^ Percentage (number of participants), Pearson chi-square. ^2^ Median (p10 and p90), Kruskal–Wallis Test.

**Table 3:** Daily food consumption by socioeconomic status (education and occupation).

	Model 1			Model 2			Model 3			Model 4		
	OR	95% CI Low	95% CI High	OR	95% CI Low	95% CI High	OR	95% CI Low	95% CI High	OR	95% CI Low	95% CI High
Raw vegetables or salad												
Low SES ^α^	0.42 ***	0.33	0.53	0.45 ***	0.35	0.57	0.45 ***	0.35	0.57	0.44 ***	0.34	0.56
Middle-low SES ^α^	0.51 ***	0.39	0.66	0.53 ***	0.41	0.69	0.53 ***	0.41	0.69	0.52 ***	0.40	0.68
Middle-high SES ^α^	0.63 *	0.47	0.84	0.63 *	0.47	0.85	0.63 *	0.47	0.83	0.63 *	0.47	0.85
Boiled or fried vegetables												
Low SES ^α^	0.47 ***	0.35	0.63	0.49 ***	0.37	0.67	0.49 ***	0.35	0.64	0.48 ***	0.36	0.65
Middle-low SES ^α^	0.55 ***	0.40	0.75	0.56 ***	0.41	0.78	0.55 ***	0.40	0.76	0.55 ***	0.40	0.76
Middle high SES ^α^	0.72	0.50	1.02	0.73	0.73	0.51	0.72	0.50	1.02	0.72	0.72	1.02
Fresh fruit												
Low SES ^α^	0.57 ***	0.48	0.68	0.61 ***	0.51	0.73	0.61 ***	0.51	0.72	0.61 ***	0.51	0.73
Middle-low SES ^α^	0.73 ***	0.60	0.88	0.75 **	0.62	0.91	0.75 **	0.62	0.91	0.75 **	0.62	0.91
Middle high SES ^α^	0.79 *	0.64	0.98	0.81	0.65	1.01	0.81	0.65	1.00	0.81	0.65	1.00
Cod or saithe liver oil												
Low SES ^α^	0.70 ***	0.59	0.83	0.75 **	0.63	0.90	0.76 **	0.64	0.91	0.77 **	0.64	0.92
Middle-low SES ^α^	0.80 *	0.66	0.97	0.84	0.67	1.02	0.84	0.69	1.02	0.84	0.69	1.02
Middle high SES ^α^	0.97	0.78	1.20	0.99	0.79	1.23	0.99	0.79	1.24	0.99	0.79	1.24
Whole-wheat bread or coarse grain												
Low SES ^α^	0.85	0.72	1.01	0.89	0.75	1.06	0.88	0.74	1.05	0.87	0.73	1.05
Middle-low SES ^α^	0.82 *	0.67	0.99	0.83	0.69	1.01	0.83	0.68	1.01	0.83	0.68	1.01
Middle high SES ^α^	0.82	0.66	1.01	0.82	0.66	1.02	0.81	0.65	1.01	0.81	0.65	1.01
Oatmeal or muesli												
Low SES ^α^	0.67 ***	0.58	0.82	0.73 ***	0.61	0.87	0.72 ***	0.60	0.86	0.71 ***	0.59	0.86
Middle-low SES ^α^	0.75 **	0.61	0.91	0.77 *	0.63	0.94	0.77 **	0.63	0.94	0.76 **	0.62	0.93
Middle high SES ^α^	0.75 *	0.60	0.94	0.76 *	0.61	0.96	0.76 *	0.60	0.95	0.76 *	0.60	0.95

SES: socioeconomic status; OR: odds ratio; CI: confidence interval. Model 1: adjusted for age and sex. Model 2: adjusted additionally for health status: BMI, exercise, smoking, alcohol, and the number of medications. Model 3: adjusted additionally for social support: living alone. Model 4: adjusted additionally for cognitive status. ^α^ Compared to high SES. * *p* < 0.05, ** *p* < 0.01, *** *p* < 0.001.

**Table 4:** Consumption of food products and drinks by socioeconomic status (education and occupation).

	Model 1			Model 2			Model 3			Model 4		
	OR	95% CI Low	95% CI High	OR	95% CI Low	95% CI High	OR	95% CI Low	95% CI High	OR	95% CI Low	95% CI High
Hot main meal, daily ^2^												
Low SES ^α^	1.07	0.87	1.31	1.15	0.94	1.42	1.15	0.93	1.42	1.14	0.92	1.41
Middle-low SES ^α^	0.95	0.76	1.19	1.00	0.80	1.25	1.00	0.80	1.25	0.99	0.79	1.24
Middle high SES ^α^	0.90	0.70	1.16	0.92	0.72	1.19	0.92	0.72	1.19	0.92	0.72	1.19
The main meal, daily ^2^												
Low SES ^α^	1.68	0.63	4.48	1.68	0.62	4.58	1.74	0.64	4.76	1.64	0.59	4.55
Middle-low SES ^α^	1.56	0.51	4.83	1.60	0.52	4.96	1.64	0.53	5.10	1.62	0.52	5.04
Middle high SES ^α^	0.54	0.10	2.81	0.54	0.10	2.81	0.55	0.11	2.86	0.55	0.11	2.86
Cultured milk products ^1,2^												
Low SES ^α^	0.61 ***	0.51	0.72	0.63 ***	0.53	0.76	0.63 ***	0.53	0.76	0.64 ***	0.53	0.76
Middle-low SES ^α^	0.69 ***	0.57	0.84	0.71 ***	0.58	0.86	0.71 ***	0.58	0.86	0.71 ***	0.58	0.86
Middle high SES ^α^	0.87	0.70	1.08	0.88	0.71	1.10	0.88	0.71	1.10	0.88	0.71	1.10
Coffee, 5 cups or more daily ^2^												
Low SES ^α^	1.68 ***	1.33	2.12	1.44 **	1.13	1.84	1.45 **	1.13	1.85	1.44 **	1.13	1.85
Middle-low SES ^α^	1.24	0.95	1.62	1.12	0.85	1.47	1.12	0.86	1.48	1.12	0.85	1.47
Middle high SES ^α^	1.18	0.87	1.59	1.09	0.80	1.49	1.10	0.81	1.49	1.10	0.81	1.49
Alcohol, currently ^3^												
Low SES ^α^	0.45 ***	0.38	0.55	0.43 ***	0.36	0.53	0.43 ***	0.36	0.53	0.43 ***	0.35	0.52
Middle-low SES ^α^	0.72 **	0.58	0.88	0.70 **	0.56	0.87	0.70 **	0.56	0.87	0.70 **	0.56	0.87
Middle high SES ^α^	0.83	0.65	1.06	0.82	0.64	1.05	0.82	0.64	1.05	0.82	0.64	1.04

SES: socioeconomic status; OR: odds ratio; CI: confidence interval. ^1^ Cultured milk products, Skyr (an Icelandic milk-curd product), or milk gruel or porridge. ^2^ Variable was dichotomized into daily consumption or more vs. less than daily consumption. ^3^ Compared to not currently consuming. Model 1: adjusted for age and sex. Model 2: adjusted additionally for health status: BMI, exercise, smoking, alcohol (not included when alcohol, currently), and the number of medications. Model 3: adjusted additionally for social support: living alone. Model 4: adjusted additionally for cognitive status. ^α^ Compared to high SES. ^α^ *p* < 0.05, ** *p* < 0.01, *** *p* < 0.001.

## Data Availability

The original contributions presented in this study are included in the article. Further inquiries can be directed to the corresponding author.
